# Thermal-Fluid–Solid Coupling—Parametrical Numerical Analysis of Hot Turbine Nozzle Guide Vane

**DOI:** 10.3390/ma14237313

**Published:** 2021-11-29

**Authors:** Marcin Froissart, Tomasz Ochrymiuk

**Affiliations:** Institute of Fluid-Flow Machinery, Polish Academy of Sciences, 14 Fiszera Street, 80-231 Gdańsk, Poland; mfroissart@imp.gda.pl

**Keywords:** heat transfer coefficient, gas turbine blade, thermal-fluid–solid coupling, turbulent Prandtl number, temperature distribution, turbine vane cooling

## Abstract

The cooling technology of hot turbine components has been a subject of continuous improvement for decades. In high-pressure turbine blades, the regions most affected by the excessive corrosion are the leading and trailing edges. In addition, high Kt regions at the hot gas path are exposed to cracking due to the low and high cycle fatigue failure modes. Especially in the case of a nozzle guide vane, the ability to predict thermally driven loads is crucial to assess its life and robustness. The difficulties in measuring thermal properties in hot conditions considerably limit the number of experimental results available in the literature. One of the most popular test cases is a NASA C3X vane, but coolant temperature is not explicitly revealed in the test report. As a result of that, numerous scientific works validated against that vane are potentially inconsistent. To address that ambiguity, the presented work was performed on a fully structural and a very fine mesh assuming room inlet temperature on every cooling channel. Special attention was paid to the options of the k−ω SST (shear-stress transport) viscosity model, such as Viscous heating (VH), Curvature correction (CC), Production Kato-Launder (KT), and Production limiter (PL). The strongest impact was from the Viscous heating, as it increases local vane temperature by as much as 40 deg. The significance of turbulent Prandtl number impact was also investigated. The default option used in the commercial CFD code is set to 0.85. Presented study modifies that value using equations proposed by Wassel/Catton and Kays/Crawford. Additionally, the comparison between four, two, and one-equation viscosity models was performed.

## 1. Introduction

To increase the efficiency and the specific work output of a gas turbine engine, it is necessary to increase the temperature and pressure of the working fluid. The direct consequence of it is a more hostile environment inside the hot gas path and life reduction of turbine stages [1]. The most loaded component is the first stage high pressure turbine blade, which is the subject of extreme centrifugal force, pressure, and temperature. To withstand such a challenging condition, these blades ought to be made with very expensive and durable nickel superalloys. However, the relentless pursuit to gain the market advantage forced engine manufacturers to employ supporting technologies like TBC (thermal barrier coating) or cooling systems. For decades various cooling techniques have been invented and patented as a result of extensive research programmes supported by gas turbine manufacturers [2]. The design target set for cooling systems is to keep blade temperature below the given threshold with the minimum consumption of expensive cooling air extracted from the compressor. The latest cooling solutions allow operation of the blade in environments much hotter (200~300 deg) than its melting point. Continuous operation in such a hostile environment is supported by the film cooling layer which forms an efficient protection layer along the blade gas washed surfaces.

Cooling air sourced in the compressor and bypassed around the combustion chamber is cold enough to ensure continuous and robust turbine blade operation. Due to the complex 3D shape, film cooling effectiveness varies significantly around the blade. To estimate it, CFD (Computational Fluid Dynamics) computation methods were developed and successfully employed in the design process. Over the years, this approach was well validated and proved to give reliable results. Haller and Camus [3] investigated aerodynamic losses for a film-cooled transonic blade. They found that suction-side film cooling rows cause much more loss than other regions. Garg and Gauler [4] used a 3D Reynolds-averaged Navier–Stokes equation to study the effect of coolant temperature and coolant to mainstream mass flow ratio on the adiabatic effectiveness of a film-cooled turbine blade. They concluded that for high flow rates on the pressure side, film effectiveness drops and temperature rises. Contrary to that, film cooling effectiveness was improved on the suction side. 

For over 30 years Computational Fluid Dynamics has been a subject of continuous improvement, so it became quite a well validated approach in solving engineering and research problems. Assuming mass, momentum, and energy conservation laws (classic mechanics) it successfully models physical aspects of real flows. However, most common CFD turbulence models are not universal, so they need to be used with caution. They were created as a result of compromise between industry (good enough result in a reasonable time) and science (focus on details). As a result of that limitation, every CFD model ought to be validated through experiment before making any conclusions. Hylton et al. [5] analytically and experimentally evaluated heat transfer distribution around the C3X internally cooled NASA vane in a straight cascade. Their work includes several test cases with varying Mach numbers, Reynolds numbers, inlet turbulence intensities, and wall-to-gas absolute temperature ratios. This extensive and open-access report has been used worldwide for decades to validate CFD models. However, coolant inlet temperature was not reported there. That missing data is the source of discrepancy among the researchers, who frequently use this vane as a validation case. For example, Laskowski et al. [6] used an inverse method to estimate these inlet temperatures. One of the main objectives of this paper is to prove that for a very fine mesh, room temperature at the coolant inlet satisfies the model validation process. An equally important aim is to present the level of simplification introduced by the rounding variable energy Prandtl number to constant value of 0.85. 

Ragab and El-Gabry [7] conducted a numerical analysis of the heat transfer over the internally cooled C3X NASA vane. They found that curvature-corrected stress transport turbulence model (SST-CC) is closer to experiment than pure SST for the pressure side temperature. Additionally, transition SST viscous model (SST_ γ_REθ) was better in capturing the transition offset, but for some regions it was worse than SST and SST-CC. Yousefi et al. [8] investigated the ribbed channel heat transfer enhancement of internally cooled C3X NASA vane. They found that longitudinal ribs in the superior configuration reduce thermal gradient and mean temperature by 25K assuring coolant flow reduction. 

There are interesting research programs containing specific C3X cooling passages optimization. Storti et al. [9] optimized the position of cooling holes using the Chimera approach. They found that better cooling performance can be achieved by shifting cooling holes closer to the suction side. Nowak and Wróblewski [10] optimized position and size of C3X cooling holes using Reduced Conjugate Heat Transfer (RCHT) algorithm. To reduce mean temperature and thermal gradient, some cooling holes were enlarged with the size reduction of others. Mazaheri et al. [11] also conducted RCHT optimization of cooling holes shape and size. They found that the shape and location of the trailing edge channel has the strongest impact to the mean temperature and thermal gradient. Karimi et al. [12] optimized C3X cooling system based on robustness improvement. They concluded, that by merging leading edge channels, blade will be much more robust in the uncertain operational conditions. 

Schmidt and Starke [13] considered blade’s temperature oscillations due to the unsteady flows. They found that wall temperature unsteadiness for the time-averaged heat load is low for typical turbine relevant frequencies. Frąckowiak et al. [14] applied an iterative algorithm to solve the inverse heat conduction problem in the multiply-connected domains. Their results revealed that this method offers many advantages for inverse problems related to the gas turbine blade cooling. Same authors [15] conducted a complex investigation containing blade’s cooling channel porosity distribution optimization. They concluded that their algorithm is applicable to the blade regions, where it is possible to remove the required amount of heat through the cooling channel. Other numerous works were published as a result of extensive analysis of turbine blade’s cooling [16,17,18,19].

Kusterer et al. [20] performed a CHT (Conjugate Heat Transfer) analysis of turbine blade with helical holes. As a result of suction and pressure side film cooling application, they achieved a reasonable match with the experiment. Esfahanian et al. [21] analyzed internally cooled turbine blade with several turbulence models using CHT method. It contained results published by Dees et al. [22] for the validation purpose. It was concluded, that for some cases V2F model is precise enough for heat transfer analysis. Han et al. [23] performed thermal analysis of internally cooled gas turbine using his own CHT code. They figured out that internal cooling systems thermal analysis could be simplified by application of coolant bulk temperature as imposed boundary condition. Takahashi et al. [24] studied gas turbine blade with ribbed cooling passages using heat transfer and friction correlation taken from the large eddy simulation performed by Watanabe [25]. They obtained a good correlation between such analysis and the experiment proving that this method is acceptable for the ribbed cooling passages. Various techniques of heat transfer enhancement in cooling channels were experimentally and numerically investigated over the years [26,27,28,29]. They included forced convection, increasing heat transfer area, phase change materials, mass flow variation and testing groves/ribs arrangements. Szwaba et al. [30] experimentally investigated aerodynamic microscale effects along the perforated plate. They found, that this technology can give a better control over the boundary layer control in the turbomachinery. Froissart et al. [31] studied jet impingement cooling case with deformed heat sink. They concluded that conical heat sink cooling effectiveness can be improved by few percent by the introduction of specific humped deformation. Kraszewski [32] performed transient fluid–solid interaction analysis to assess the spherical Y-pipe load during an ultra-fast start-up and shutdown. That approach helped to capture the complex interaction between fluid and solid states. 

The best available numerical model is named the Direct Numerical Simulation (DNS), which lets to model complex flow structure with excellent accuracy [33]. Nevertheless, it is not widely used due to the very high computation cost. That is the reason why URANS (Unsteady Reynolds-Averaged Navier–Stokes) models are still the subject of extensive development programs. The side effect of using them is the necessity of additional terms introduction in the governing equations in order to achieve a “closure” for the unknowns. Currently they are hundreds of hypotheses on how to do that, so in each case it is the subject to the individual decision. Each method of turbulence model “closure” is only an approximation, so inevitably some part of real-flow physics is omitted [34]. As a result of that, models were grouped according to the flow classes. The most popular and universal ones are good enough at the global level, but differences between them are clearer when focusing on details. Nevertheless, using URANS models is a way of an effective compromise between the computation time, robustness, and accuracy for quite a wide spectrum of flow phenomena. More computational expensive alternative is LES (Large Eddy Simulation) model, which appears in some advanced research studies. According to the experience and available data, RANS (Reynolds-Averaged Navier–Stokes) and URANS phenomenological turbulence models is a verified and efficient solution in gas turbines flow modelling—especially in the initial, hottest turbine stages. In simplified cases like cold turbine flows without the heat transfer, the simplest models like Spalart-Allmaras of k-epsilon give satisfactory results in terms of flow modelling. Special attention to the turbulence model selection ought to be paid to properly capture heat transfer phenomena between the aerofoil and hot gas patch. 

Turbulent heat flux is an example of parameter difficult to “close” phenomenologically and even more tough to validate by measurement. Furthermore, it is treated as a lower priority factor, because the key modelled phenomenon is the turbulent transport of momentum. That is the reason why the heat transfer along the stream (membrane cooling) is modelled as a turbulent heat transfer, which is “closed” according to the gradient hypothesis. It is based on auxiliary parameter characterizing turbulent diffusive heat transfer (analogical to the turbulent viscosity), which links turbulent energy flux with mean temperature gradient. 

The presented paper is arranged in four sections. The first one is an introduction, which place obtained results in the scientific context. Second one contains problem statement, which includes geometry, model, governing equations, reference experiment, and boundary conditions. Third section is focused on the results and discussion, which is about the viscous model comparison, model validation and modification of the turbulent Prandtl number. The last section contains conclusions regarding the impact of selected viscous models and heat transfer coefficient distribution along the external surface of analyzed C3X nozzle guide vane. 

## 2. Problem Statement

NASA C3X internally cooled vane is one of the most popular test cases, widely accepted and used among scientific community for decades. The report containing experimental setup, detailed geometry, boundary conditions and gauge readings was published in 1983 by Hylton et al. [5]. Its main objective was to assess the capability of available modelling methods for predicting heat transfer characteristic of 2D flow field. Experimental and analytical results were structured to improve the gas turbine design process. 

The experimental measurements were taken from the middle vane in the three-vane cascade. The moderate temperature (~800 K) and steady state conditions were chosen to improve the measurement accuracy. The independent input parameters were Mach number, Reynolds number, turbulence intensity, and wall-to-gas temperature ratio. 

**Figure 1:**
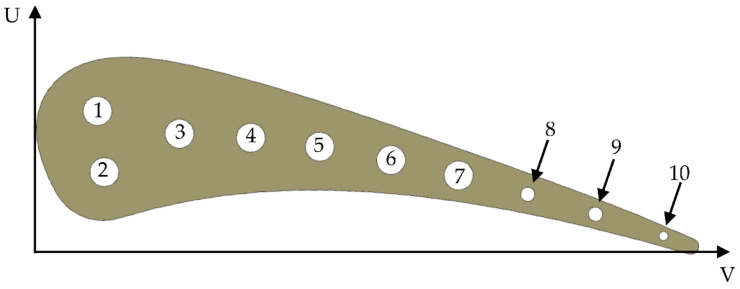
C3X section with cooling holes’ locations.

Figure 1 presents a section of aerofoil with 10 straight radial cooling holes. Holes from three to ten are positioned along the camber line. Due to the high sectional area and high heat flux at the leading edge, the first two cooling holes were positioned on both sides of the camber line. In the principal assumption this was a 2D test, so the section is constant through the entire vane’s height (76.2 mm). Measurements are taken at the midspan to minimize the impact of shroud and hub’s recirculation. 

Table 1 summarizes the position of each cooling hole in the local coordinate system. Inlet temperature in not stated in the report [5], but it is suggested, that inlet temperature is constant for all holes. Report says: “the cooling holes of each of the outer two slave vanes of each cascade were supplied from a common plenum, whereas each hole in the test vane (at the center position) was supplied from a separate, metered line”. In another place report says: “Tw/Tg levels were varied by controlling the vane coolant flow rate”. Report does not mention about controlling inlet temperature, which is not required in the presence of the flow rate control. Additionally, in the absence of inlet temperature information, the default option should be room temperature (293.15 K), because there is no point to heat the coolant in the presence of flow rate control system. Based on this logic, coolant at room temperature was taken for further analysis. 

**Figure 2:**
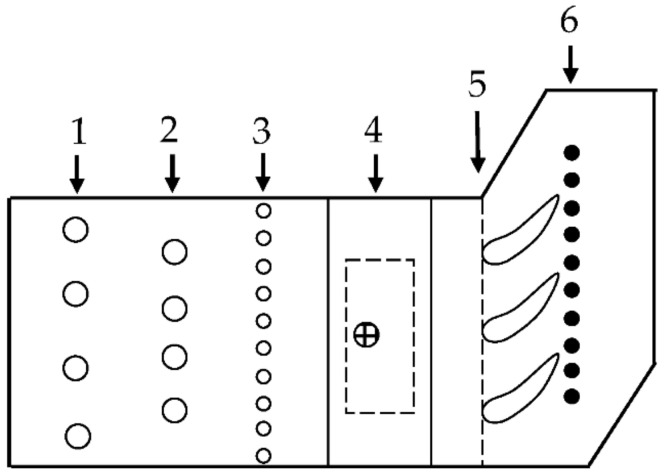
C3X test facility schematic.

As presented on Figure 2, experimental setup contained a cascade of three internally cooled straight vanes, turbulence augmentation rods (station 1), total pressure and total temperature racks (station 2), inlet static pressure taps (station 3), laser Doppler anemometry measurement volume (station 4) and exit static pressure taps (station 6).

In total 18 different configurations were tested. They varied with Mach exit number, exit Reynolds number, inlet turbulence intensity and ratio between wall and gas temperature (Tw/Tg). Case 158 was taken for further analysis and validation (Table 2).

### 2.1. Geometrical Setup

**Figure 3:**
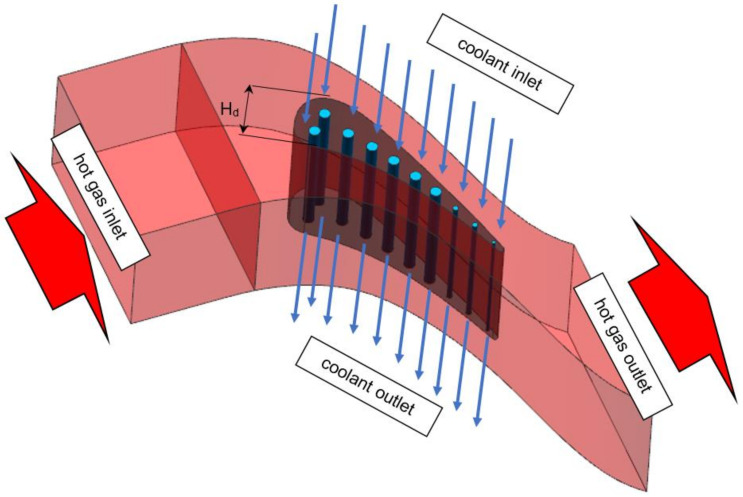
Geometry taken for analysis with flows’ directions (periodic boundary condition).

Figure 3 presents geometry taken for meshing and analysis. It contains one solid domain and 11 fluid domains. Each of ten cooling channels is a straight cylinder positioned inside the aerofoil [5]. External hot gas path domain has got one inlet and one outlet. Side surfaces were profiled to match each other helping with cyclic boundary condition application. Upper and lower planes are flat to model the actual tested cascade. Boundaries between domains perfectly match each other to help with meshing and interface contact application. Due to the fact, that model is symmetric and validation process is focused at the midspan, it does not matter if cooling passages are fed from the top or from the bottom. Hot gas path hydraulic diameter H_d_ taken for analysis is equal to 34 mm (Figure 3).

### 2.2. 3D Mesh Generation

**Figure 4:**
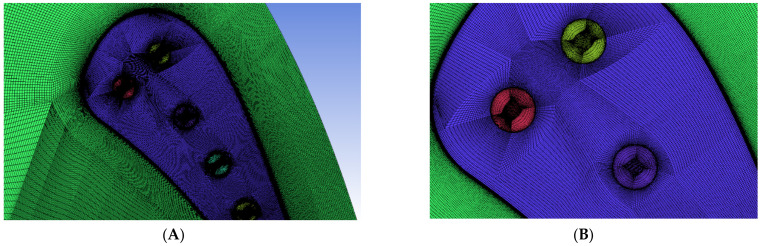
Mesh refinement around leading edge, (**A**) half view, (**B**) front view.

**Figure 5:**
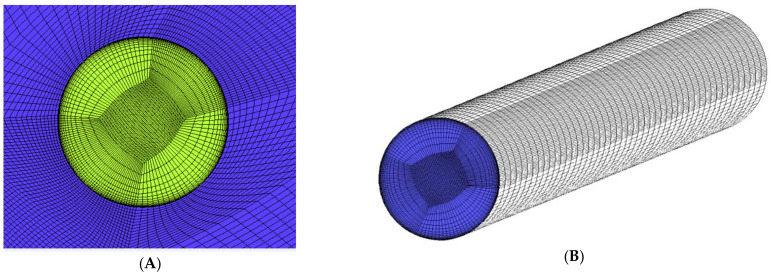
Mesh refinement of the first cooling hole, (**A**) inlet view, (**B**) interface view.

Geometry presented on Figure 3 was meshed using blocking method to achieve fully structured mesh with matching faces. As a result of that, analyzed mesh contains only hexahedral elements with dense inflation layers around the gas washed surfaces. The mesh quality is one of the key parameters of trustworthy and validated model. The presented case model contains 13.5 million elements and 14 million nodes. Additionally, mesh between solid and fluids is consistent across every interface, so heat transfer coefficient is not affected by the mesh mismatch interpolation process. According to the experience, mesh mismatch on such curved surfaces like the trailing edge cooling hole introduces significant interpolation errors. Figure 4 presents a mesh sample around the leading edge. It is clear that mesh blocks match each other at interfaces, maintaining inflation layer and a consistent mesh size. Figure 5 is focused on the example of a cooling hole, in which mesh is based on an o-grid (meshing method available in ICEM software designed to apply boundary layer inflation) and contains regular element layers in depth. Every hole in the modelled C3X blade is meshed in the same manner.

One of the most common metrics of mesh quality is $Y^{+}$ parameter, defined as:

(1) $$Y^{+}=\frac{yu_{Τ}}{\nu }$$

where $u_{Τ}$ is the so-called friction velocity, y is the absolute distance from the wall, ν is the kinematic viscosity. 

**Figure 6:**
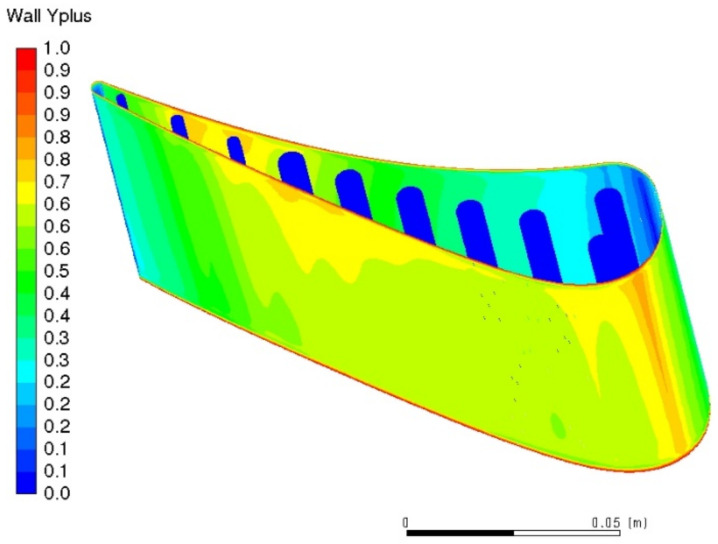
$Y^{+}$ meshing assessment factor plot.

Figure 6 presents a plot containing $Y^{+}$ value for all gas washed surfaces—including the highly curved critical region at the suction side. In the very fine mesh used for the following analyses, $Y^{+}$ value is smaller than one, so mesh is deemed acceptable. 

### 2.3. Governing Equations

Because this exact Navier–Stokes equation is very computationally expensive, applied viscosity models are based on a Reynolds-averaged Navier–Stokes (RANS) equations shown below (Equations (2) and (3)).

(2) $$\frac{\partial \rho }{\partial t}+\frac{\partial }{\partial x_{i}}\left(\rho u_{i}\right)=0$$

(3) $$\frac{\partial }{\partial t}\left(\rho u_{i}\right)+\frac{\partial }{\partial x_{j}}\left(\rho u_{i}u_{j}\right)=-\frac{\partial p}{\partial x_{i}}+\frac{\partial }{\partial x_{j}}\left[\mu \left(\frac{\partial u_{i}}{\partial x_{j}}+\frac{\partial u_{j}}{\partial x_{i}}-\frac{2}{3}\delta_{ij}\frac{\partial u_{l}}{\partial x_{l}}\right)\right]+\frac{\partial }{\partial h_{j}}\left(-\rho \bar{u\prime_{i}u\prime_{j}}\right)$$

They were derived from the exact form of Navier–Stokes equation by decomposing components into the time-averaged and fluctuating components. In particular, velocity can be decomposed according to Equation (4).

(4) $$u_{i}=\bar{u_{i}}+u\prime_{i}$$

where:

$\bar{u_{i}}$—mean velocity component [m/s]

$u\prime_{i}$—fluctuating velocity component [m/s]

The additional stress term at the end of Equation (3) $\left(-\rho \bar{u\prime_{i}u\prime_{j}}\right)$ is unknown, so it needs to be closed somehow. To help with it, RANS viscosity models are used in the CFD (Computational Fluid Dynamics) codes. In this paper, results are mainly focused on the k−ω SST (Shear Stress Transport) turbulence model, which applies k−ω model close to the wall and k−ε model in the freestream. Between those two regions models are blended, so k−ω SST model is a good compromise between those two models. Transport equations for SST k−ω model are given below:

(5) $$\frac{\partial }{\partial t}\left(\rho k\right)+\frac{\partial }{\partial x_{i}}\left(\rho ku_{i}\right)=\frac{\partial }{\partial x_{j}}\left(\Gamma_{k}\frac{\partial k}{\partial x_{j}}\right)+\tilde{G_{k}}-Y_{k}+S_{k}$$

and

(6) $$\frac{\partial }{\partial t}\left(\rho \omega \right)+\frac{\partial }{\partial x_{i}}\left(\rho \omega u_{i}\right)=\frac{\partial }{\partial x_{i}}\left(\Gamma_{\omega }\frac{\partial \omega }{\partial x_{j}}\right)+G_{\omega }-Y_{\omega }+D_{\omega }+S_{\omega }$$

The concept of numerical modelling of cooling systems requires application of “hot” turbulence models, so it is desirable to model both flow’s turbulent parameters: momentum and heat. In work [11] the turbulent heat transfer concept was used to improve the accuracy of turbine blades’ membrane cooling prediction. That was concluded by the comparison with the previous author’s research results [35,36,37] which focused on the turbulent heat transfer factor near the wall. As mentioned above, the most common modelling approach is based on turbulent momentum exchange and species diffusion rather than turbulent heat flux $\vec{q_{t}}$. The most common solution is the conversion of turbulent momentum exchange equation into the turbulent heat transfer one shown below.

(7) $$\vec{q_{t}}=\alpha_{t}\nabla T=\frac{c_{p}\mu_{t}}{Pr_{t}}\nabla T$$

where:

$\vec{q_{t}}$—heat flux [W/m^2^]

$c_{p}$—specific heat [J/(KgK)]

$\mu_{t}$—turbulent viscosity [kg/m∙s]—function of two parameters κ, ε governing the turbulent momentum exchange

$Pr_{t}$—turbulent Prandtl Number [-] 

T—temperature [K]

Above equation is analogical to the molecular heat conduction equation of single gas ingredient.

(8) $${\vec{q}}_{\left(k\right)}=\alpha_{\left(k\right)}\nabla T_{\left(k\right)}=\frac{c_{p\left(k\right)}\mu_{\left(k\right)}}{Pr_{\left(k\right)}}\nabla T_{\left(k\right)}$$

where:

$\vec{q_{\left(k\right)}}$—heat flux [W/m^2^]

$c_{p\left(k\right)}$—specific heat [J/(KgK)]

$\mu_{\left(k\right)}$—molecular viscosity [kg/m∙s] 

$Pr_{\left(k\right)}$—Prandt Number [-] 

T—temperature [K]

The description of $\vec{q_{t}}$ in a phenological and statistical sense is the worst described element of turbulence theory. From the phenological point of view, the algebraically “closure” modelling could be successfully replaced by the differential evolutionary equations.

(9) $$\vec{q_{t}}{}^{\circ}+\frac{1}{\tau_{q}}\vec{q_{t}}=f\left(\cup ,\nabla \cup ,\partial_{t}\cup \right)$$

where:

$\cup =\left\{\rho ,\rho \vec{v},\rho e,\ldots \right\}$—represents the whole vector of conservative variables 

∇∪—dimensional change of vector U

$\partial_{t}\cup$—time change of vector U 

$\vec{q_{t}}{}^{\circ}$—heat flux time derivative is independent from the coordinate system 

$\tau_{q}$—relaxation time

The simplest approach to solve Equation (8) is to apply constant turbulent Prantl number equal to 0.9 (boundary layers) or as low as 0.5 for free shear flows [38]. Constant value of it assumes an analogy between the turbulent heat and momentum transfer. More precise solution is based on the algebraic (zero-equation) model. Many of them link eddy diffusivity with turbulent viscosity and a turbulent Prandtl number (Equation (10)).

(10) $$\bar{\rho }\alpha_{T}=\frac{\mu_{T}}{Pr_{T}}$$

Equation (11) was developed by Wassel and Catton, where coefficients are equal to: $C_{1}$ = 0.21, $C_{2}$ = 5.25, $C_{3}$ = 0.20, $C_{4}$ = 5.00.

(11) $$Pr_{T}=\frac{C_{3}}{C_{1}Pr}\left[1-exp\left(\frac{-C_{4}}{\left(\mu_{T}/\mu_{L}\right)}\right)\right]{\left[1-exp\left(\frac{-C_{2}}{\left(\mu_{T}/\mu_{L}\right)Pr}\right)\right]}^{-1}$$

Another, more complex algebraic formula to calculate turbulent Prandtl numbers was defined by Kays and Catton (Equation (13)), which is linked to the turbulent Peclet number (Equation (12)).

(12) $$Pe_{T}=\left(\frac{\mu_{T}}{\mu_{L}}\right)Pr$$

(13) $$Pr_{T}={\left\{\frac{1}{2Pr_{T\infty }}+\frac{CPe_{T}}{\sqrt{Pr_{T\infty }}}-{\left(CPe_{T}\right)}^{2}\left[1-exp\left(\frac{-1}{CPe_{T}\sqrt{Pr_{T\infty }}}\right)\right]\right\}}^{-1}$$

where:

$Pe_{T}$—turbulent Peclet number [-]

$\mu_{T}$—turbulent viscosity [kg/m∙s]

$\mu_{L}$—molecular viscosity [Pa s]

Pr—Prandtl number [-]

$Pr_{T}$—Prandtl number far from the wall [0.85]

C—constant [0.3]

### 2.4. Boundary Conditions

The geometry and flow alignments taken to the analysis are presented on Figure 3. Single 3D C3X aerofoil was modelled with ten internal cooling passages and one external hot gas patch domain. Inlet and outlet parameters of main flow path are listed in Table 2, which is directly based on the NASA report [5].

where:

$P_{1}$—absolute inlet pressure 

$P_{2}$—absolute outlet pressure 

*T_1_*—inlet temperature 

$M_{1}$—inlet Mach number 

$M_{2}$—outlet Mach number 

*Tu*—inlet turbulence intensity 

H_d_—inlet hydraulic diameter (hot gas path)

The only exception is the absolute outlet pressure P_2_, which was figured out according to isentropic relation shown on Equation (14).

(14) $$P_{2}=P_{1}{\left(1+\frac{\gamma -1}{2}M_{2}{}^{2}\right)}^{\frac{-\gamma }{\gamma -1}}$$

where:

$P_{1}$—absolute inlet pressure [Pa]

$P_{2}$—absolute outlet pressure [Pa]

 γ—specific heat ratio [-]

$M_{1}$—inlet Mach number [-]

$M_{2}$—outlet Mach number [-]

**Figure 7:**
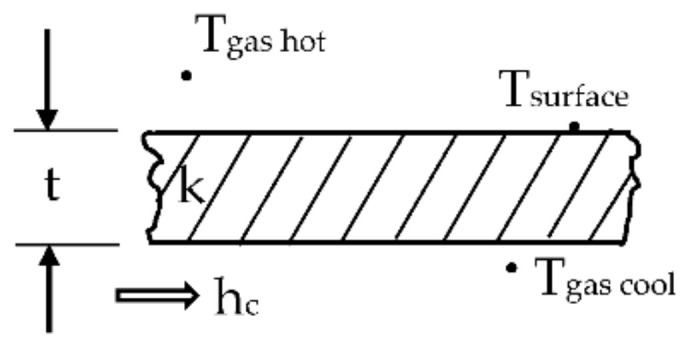
C3X vane input parameters measurement locations.

According to the NASA report [5], wall to gas temperature ratio was controlled by the coolant flow rate. Average temperature and coolant flow rate values used for analysis were taken from NASA report [5] (run number 158, case 4321, page 195). It states exact coolant rate for each cooling hole and an average temperature for every cooling hole. It was calculated assuming a linear temperature rise through the vane cooling hole. Figure 7 suggests that temperature measurements were taken at points close to the boundary layer. 

Vanes are made from ASTM 310 stainless steel, which has a relatively low thermal conductivity to minimize the error introduced by the grooves. Physical parameters of steel were taken according to Yousefi et al. [8]:

density ρ = 7900 [kg/m^3^]

specific heat c_p_ = 586.15 [J/(kgK)]

thermal conductivity k(T)=0.020176T + 6.811 [W/(mK)]

Real-gas-air physical properties were used for hot and cold flows using NIST (National Institute of Standards and Technology) database. 

### 2.5. Analysed Models

Table 3 summarizes 11 viscosity models’ variations taken for analysis in the commercial software. First one is a reference case, which was validated against the experiment. It used k−ω SST viscous model with all additional options enabled (marked by “+” sign in the table). Additionally, this case used a default energy Prandtl number equal to 0.85.

Models from two to four were used for the energy Prandtl number study. Model number two assumed a constant Prandtl number at the upper limit equal to one. Model three assumed a variable Prandtl number according to Wessel and Catton [38] formula presented on Equation (11). That was obtained by application of UDF (user defined function) file. Model four was similar to three, but Prandtl number was varied according to Kays and Crawford [38] Equation (13). 

Models from five to eleven are different in terms of applied viscus model and activated options. Model five was chosen as an alternative to k−ω SST. This four equations model was used by some scientists in the aerodynamics studies. On the other side is model eleven based on single equation Spalart-Allmaras theory. It was chosen to check its applicability in the analysis of turbomachinery. Models from six to ten are alternations of reference model one. They were chosen to answer the question about their significance during the model validation problem. 

### 2.6. Numerical Accuracy

The entire 3D cyclic thermal-FSI model has been discretized by a hexagonal mesh, steeply refined in the normal surface direction. Performed sensitivity study proved that further mesh refinement does not change the computational results significantly. It has been assumed that the channel surface is smooth and homogeneous. Wall function $Y^{+}$ below unity has been implemented for all surfaces (Figure 6). The standard SIMPLE (semi-implicit method for pressure-linked equations) method was employed for pressure-velocity coupling. Second-order accuracy methods were employed for spatial discretization, with Least Squares Cell Based method applied for gradient of components of vector of conservative variables. Iterative convergence was achieved with three orders of magnitude decrease in the normalized residuals for each equation solved (energy equation criteria was achieved with six orders of magnitude). The most challenging residual value was continuity. Double precision numerical values were used for analysis All results were obtained from ANSYS Fluent 19.1 code. Furthermore, reference CFD model was validated against experimental data [5].

Regarding the truncation error in the numerical simulation, it is assumed that it is the difference between the exact value of the partial derivative equation and its representation of the finite difference scheme. Presented analyses are based on the reference model validated against the experiment [5], where acceptable agreement was achieved. Numerical artificial diffusivity or viscosity originally was a byproduct of developing poor and lower order differencing schemes. High-order methods used for presented analyses have naturally less numerical diffusivity and viscosity than low-order methods, so this impact is deemed negligible. 

## 3. Results and Discussion

### 3.1. Validation 

**Figure 8:**
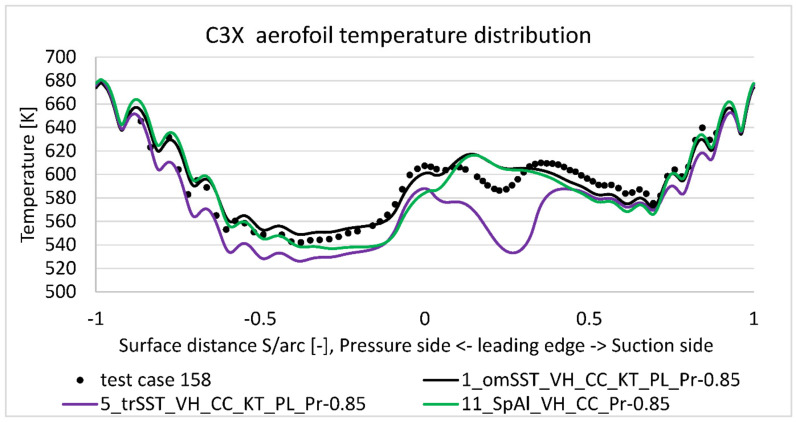
Predicted vane external surface temperature at midspan versus experimental results (measurement uncertainty is ±11 K).

Figure 8 presents midspan temperature distribution obtained for run 158’s condition summarized in Table 3 (model 1, 5, and 11). Horizontal axis contains normalized distance along pressure side (negative values) and suction side (positive values) with leading edge point at position zero and trailing edge point at position minus one and one. Negative values are used for consistency with NASA report [5]—their meaning is to mark pressure side region. Overall k−ω SST model matches quite well to the experimental data, with the maximum discrepancy equal to 20 deg. at the most curved part of suction side (S/arc ≈ 0.2). This was expected to some extension, as due to the significant pressure gradients, suction side peak is the most difficult region to properly capture with k−ω viscus models. Average temperature discrepancy for a nominal case (model 1) is estimated at 10 deg., which is acceptable value from the validation point of view. On the other side, surface temperature measurement used a well-developed technique, utilizing calibrated reference junctions, thermocouple wire calibrations, precision volumeter, and computerized temperature/millivolt table lookups. Thanks to that, the measurement temperature uncertainty is estimated as low as ±1 deg. [5]. Note that Figure 8 reference temperature distribution was generated with k−ω SST model with certain options activated: viscous heating (VH), curvature correction (CC), Production Kato-Launder (KT), and Production limiter (PL). 

To address k−ω model limitation affecting aerofoil’s overall drag and lift characteristics, transition SST ($\gamma -{\bar{Re}}_{\theta ,t}$) four equations viscus model was developed (model 5). As presented on Figure 8, it better captures the suction side temperature distribution, but underpredicts it by 45 deg., which is significantly worse than k−ω SST model. For a more comprehensive comparison, it was decided to include Spalart–Allmaras’s equation result (model 11) on Figure 8. In terms of average error, it predicts temperature distribution better than Transition SST model but worse than k−ω SST. In terms of distribution, similarly to k−ω SST model, it does not predict properly the negative peak at the most curved suction side (point 0.2). As a conclusion from Figure 8, k−ω SST viscosity model gives the best matching to the experimental data, so it was taken for further analysis. 

**Figure 9:**
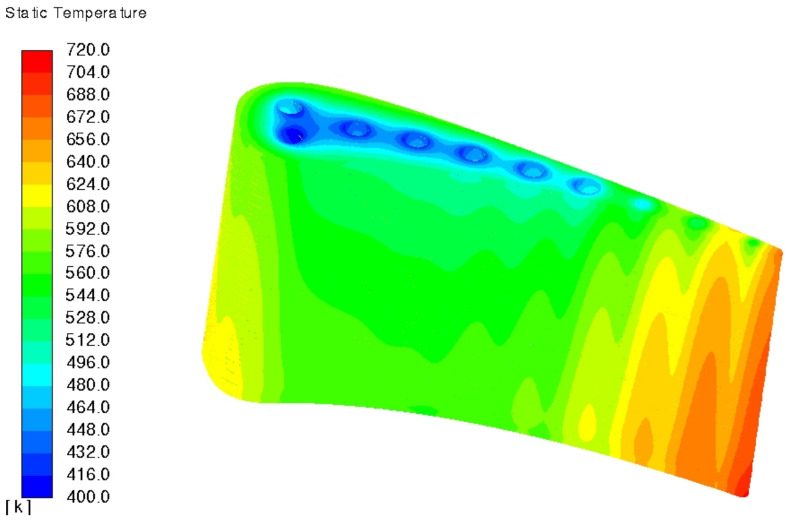
Pressure side temperature plot of model 1, run 158 (k−ω SST).

**Figure 10:**
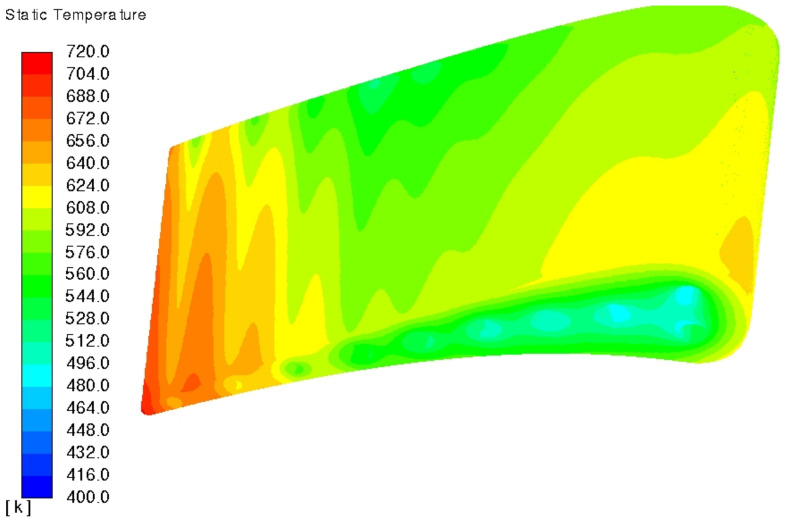
Suction side temperature plot of model 1, run 158 (k−ω SST).

Figure 9 and Figure 10 present 3D temperature plot which matches Figure 8 at the midspan position. At stated before, C3X aerofoil is straight with constant section shape and area, which is required for 2D conclusions made in NASA report [5]. As a result of that, any radial temperature variation is a result of internal cooling process—coolant temperature steadily grows along the flow path, so its cooling ability is higher at the inlet that at the outlet. In other words, cooling efficiency drops with the lower coolant/metal thermal gradient. NASA report [5] does not state the direction of coolant flow, as it does not matter for the 2D analysis at the midspan. However, that insignificant ambiguity could lead to the mirror transformed plot when comparing results with other literature positions. 

**Figure 11:**
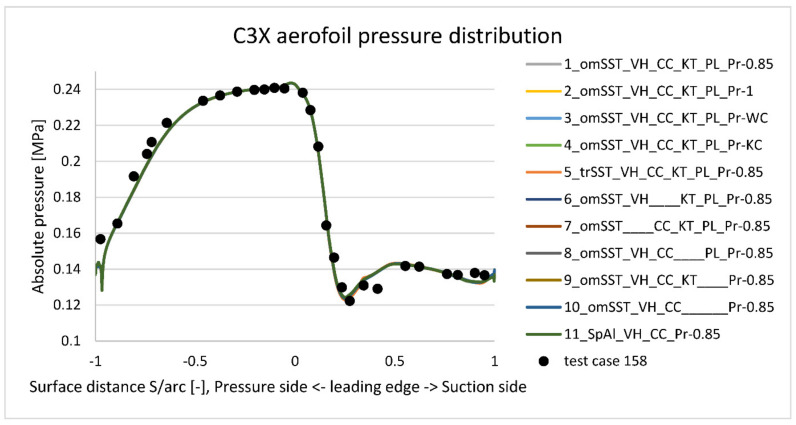
Predicted vane external absolute pressure at midspan versus experimental results (measurement uncertainty is ±700 Pa).

Figure 11 presents absolute pressure distribution plot at the midspan external surface obtained with 11 viscus models summarized in Table 3. Two main conclusions can be drawn out of it. First and main one is that k−ω SST reference viscus model predicts well absolute pressure along the midspan external surfaces. Especially positive and negative peaks are well predicted. Second important conclusion is that all eleven viscus models taken into account (Table 3) give consistent pressure plot—results are so close, that all lines lie on top of each other. 

**Figure 12:**
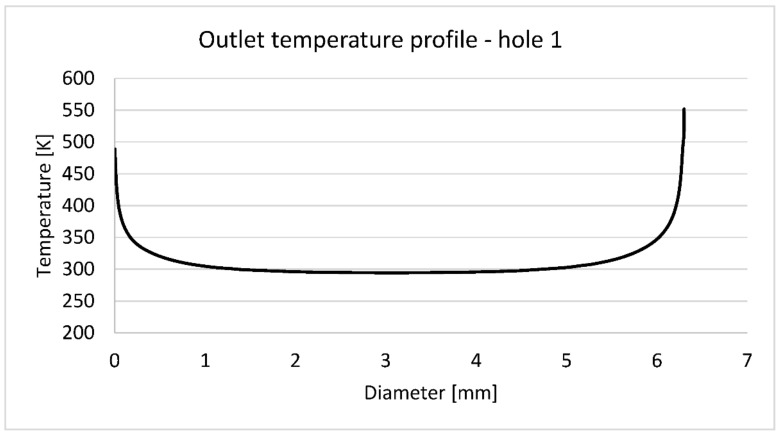
Outlet temperature distribution along the diameter highlighted on Figure 13.

**Figure 13:**
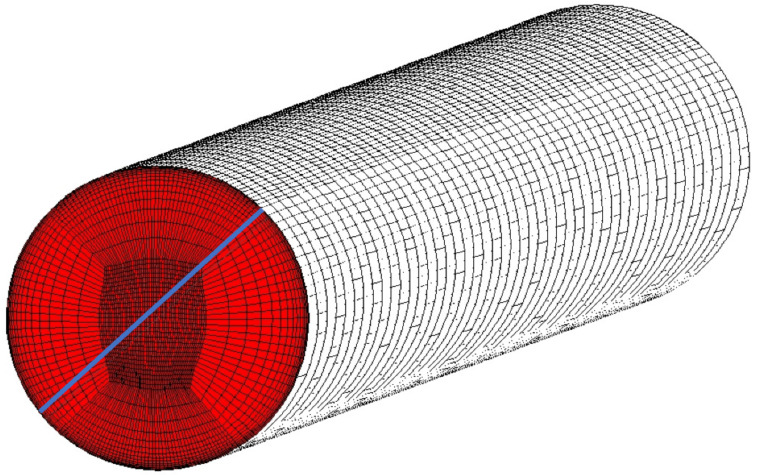
First cooling hole discretization with the linear section.

Figure 12 contains outlet temperature profile along the diameter of the first cooling hole presented on Figure 13. As expected, steep temperature gradient occurs in the wall boundary layer sourced from the NASA report [5] states that an inlet/outlet average temperature for the first cooling hole is 358.14 K, so the outlet temperature is 423.13 K (assuming 293.15 inlet temperature). Unfortunately, Figure 7 does not show exactly the position of coolant thermocouples, but 423.13 K is close enough to the wall to accept it as a part of validation process. 

### 3.2. Grid Independency

Mesh density is an important parameter, which could significantly skew obtained results. Every mesh is only an approximation of the complex reality which is anchored at the molecular level. In most classic cases finite-volume method reduces the real flow complexity to make it acceptable from the computational time point of view. The grid density selection is a form of compromise between result’s accuracy and the computational time. In the extreme case, too coarse mesh can cause a computational divergence leading to the analysis error. 

**Figure 14:**
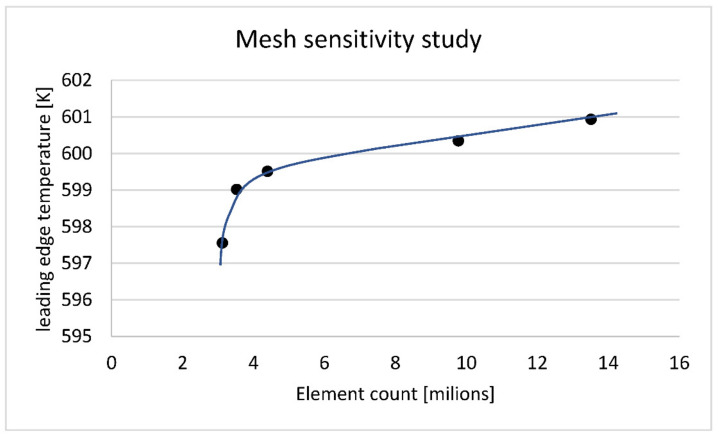
Mesh refinement impact to the midspan leading edge temperature.

Figure 14 presents the mesh density sensitivity study. Each point represents a separate model with different element (node) count. All cases are full hexagonal meshes based on the same block division like nominal model (13.5 milion elements). The element count difference is uniformly distributed within the entire model. Details of the reference mesh and block topology is shown of Figure 4 and Figure 5. The continuous line on Figure 14 has a typical shape for mesh size study. It contains two linear parts and an arc. The middle point of an arc (3.5 milions elements) marks critical point, below which mesh error grows quickly. Although the line between 3.5 and 13.5 milions is not perfectly horizontal, the mesh needs to be doubled to get an effect of a one-degree-hotter leading edge. That level of change is considered benign, so 13.5 milions element mesh was accepted for further analysis. 

### 3.3. Turbulent Prandtl Number Impact

**Figure 15:**
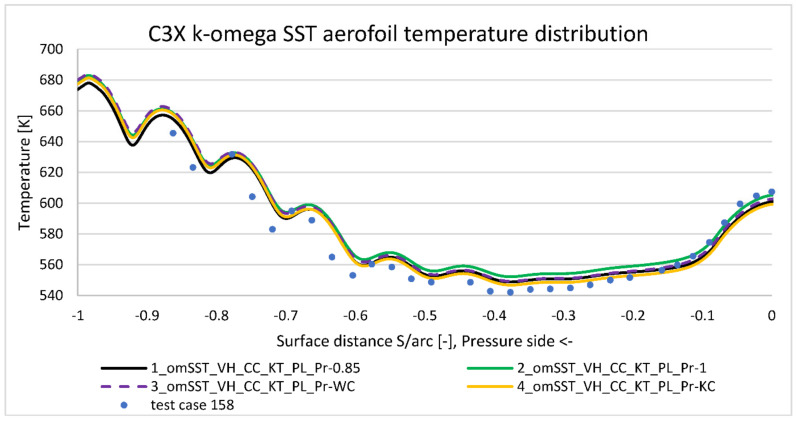
Pressure side temperature distribution comparison according to Prandtl number correction.

**Figure 16:**
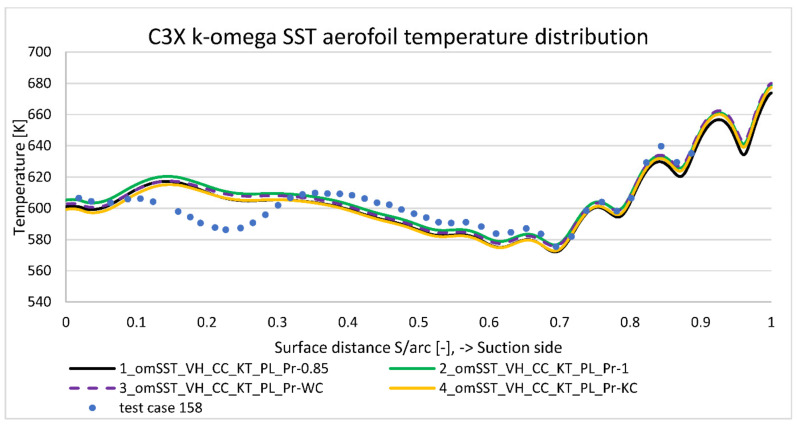
Suction side temperature distribution comparison according to Prandtl number correction.

Energy Prandtl number is an “artificial” parameter, which accounts for additional thermal conductivity due to the flow turbulence (Equation (10)). By default, it is assumed as a constant value equal to 0.85. Figure 15 and Figure 16 present turbulent Prandtl number impact to the temperature distribution along the pressure and suction sides. The definition according to the Kays and Crawford (Equation (13)) slightly drops the temperature for the pressure side ($Pr_{T}$ below 0.85) and leading edge; it is neutral for the suction side ($Pr_{T}$ close to 0.85) and adds temperature for the trailing edge area ($Pr_{T}$ close to 1.00). That improves slightly temperature prediction on the pressure side, but it has no significant impact to suction side.

In contrast to that, Wassel and Catton (Equation (11)) correction is neutral on the pressure side, but improves prediction on the suction side. Both correction methods increase temperature prediction in the trailing edge area with the $Pr_{T}$ number closer to 1. Nevertheless, turbulent Prandtl number correction affects predicted temperature by a maximum of a few degrees, which explains why constant value application is deemed acceptable in the turbomachinery applications. 

### 3.4. k−ω Viscosity Model Options Study

**Figure 17:**
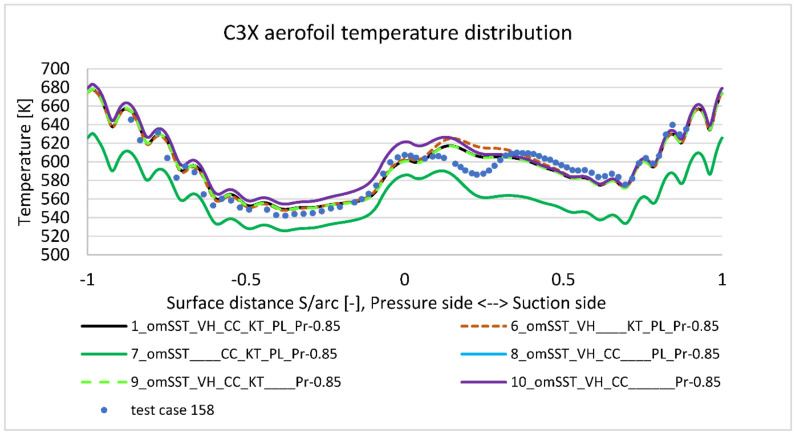
k-omega SST options temperature impact study.

Due to the fact, that every RANS viscosity model is only an approximation of real flow complex physics, researchers proposed numerous modifications to improve existing models. k−ω SST viscous model available in used commercial software is also extended by most popular additional options. The presented paper takes into account four of them (Table 3): “viscous heating” (VH), “curvature correction” (CC), “production limiter” (PL) and “production Kato-Launder” (KL). Figure 17 compares them and their contribution to the model accuracy. Obtained results revealed, that the strongest impact to the temperature prediction has “viscous heating” option (model 1 vs. 7), as it moves entire graph upwards by 40 degrees. “Curvature correction” (model 1 vs. 6) improves matching around the most curved suction side, dropping graph closer to test values by maximum 7 degrees. Lack of “Production limiter” and “production Kato-Launder” (model 1 vs. 10) options have the highest impact on the leading edge (~20 K overshoot). Interestingly, lack of “Production limiter” or “production Kato-Launder” has minor impact to temperature, which proves the interaction between those options. 

## 4. Conclusions

The presented work is based on NASA C3X vane, which was extensively tested and reported by Hylton et al. [5]. Run 158 was taken for analysis from the list of 18 test points controlling inlet turbulence intensity, exit Mach number, exit Reynolds number, and wall-to-gas temperature ratio. Results included in this paper are based on 3D thermal FSI (Fluid–Solid Interaction) model successfully validated against NASA experiment. C3X vane case is extremely popular among researchers due to the high-quality results easily available in the public domain. However, not all parameters were reported by NASA—especially coolant inlet and outlet temperatures were omitted. Instead, average temperature for each cooling hole was included, because report was focused on 2D analysis of midspan section. Currently (few decades later) in the era of 3D models, lack of inlet temperature can cause confusion and discrepancies among researchers. In the presented paper, room temperature (293.15 K) was taken as a coolant inlet temperature. That choice was supported by extensive parameter analysis run of a very fine, structural mesh.

Finite Element mechanical analysis based on presented thermal analysis is a step required in every design process. In the presented case, three-dimensional temperature distribution will lead to the thermal fight between hot and cold regions. Validated thermal map of nozzle guide vane is also extremely important [39] to predict thermal loads between vane and adjacent components made from other materials characterized with higher or lower thermal expansion coefficients.

Another key point of presented results is the comparison study between chosen viscus models. In literature, turbomachinery CFD analysis is most often analyzed with a two-equation k−ω SST or a four-equation Transition SST ($\gamma -{\bar{Re}}_{\theta ,t}$) model. However, each of these models have additional options available, which noticeably impact temperature distribution on the aerofoil. Without the full model information, it is difficult to replicate published results. The presented paper is focused on four options available for k−ω SST: Viscus Heating (VH), Curvature Correction (CC), Production Limiter (PL), and Production Kato-Launder (KL). The best result was achieved after the application of all of them. In such case, temperature distribution along aerofoil’s external surface is within few degrees to the experiment for most regions. The only exception is the highly curved suction side, where k−ω SST model does not predict local drop of temperature overestimating it by 20 deg. The highest impact from these four options has viscus heating, which for some regions drops predicted temperature by 40 degrees. As expected, curvature correction has the strongest impact to the most curved suction side region, where it drops temperature by about 7 degrees closer to the test. Disabling production limiter or production Kato-Launder separately has no impact to the temperature plot. A noticeable effect occurs when both production options are disabled. In such case, predicted temperature is consistently higher, with the strongest impact on leading edge point (20 deg.). No impact was noticed at the pressure distribution prediction, which is very close to the experimental data. 

Apart from the k−ω SST options study, high level comparison was made between four-equation Transition SST ($\gamma -{\bar{Re}}_{\theta ,t}$) model, two-equation k−ω SST and one-equation Spalart-Allmaras. It was found that the Transition SST model is better in predicting the temperature distribution along the external surface, but it systematically underpredicts it by about 20 degrees. In the most curved suction surface region, this discrepancy is as high as 45 degrees. For comparison purpose, a simpler one-equation Spalart-Allmaras was taken into account. In terms of average temperature mismatch, it is much better than complex Transition SST model. It differs from k−ω SST model especially in the leading-edge region, but on average the prediction error is about 10 degrees. That is slightly worse than k−ω SST model, which predicts temperature at the mean error of 5 degrees. No impact was noticed at the pressure distribution prediction, which is very close to the experimental data. 

Additional parameter analyzed in the presented work is the impact of the turbulent Prandtl number ($Pr_{T}$) definition. As a default parameter it is equal to 0.85 in the used commercial software. By using UDF (user defined function), turbulent Prandtl number was modified according to Wassel/Catton (WC) and Kays/Crawford (KC) equation. As a reference line, additional model with $Pr_{T}$ equal to one was analyzed. Results show, that both definitions affect temperature by about one degree, which explains why constant value is an acceptable default option in the commercial software. In general, Kays and Crawford correction improves temperature prediction in the pressure side, whereas Wassel and Catton is better for leading edge and suction side. Both models give similar correction at the trailing edge, which is close to the constant $Pr_{T}$ value of one. 

## Figures and Tables

**Table 1:** C3X cooling holes’ position, diameters and assumed inlet coolant temperature.

Hole No. [-]	U [cm]	V [cm]	Diameter [cm]	Inlet Temperature [K]
1	2.377	1.311	0.63	293.15
2	1.057	1.534	0.63	293.15
3	1.981	3.119	0.63	293.15
4	1.981	4.674	0.63	293.15
5	1.869	6.182	0.63	293.15
6	1.666	7.747	0.63	293.15
7	1.412	9.235	0.63	293.15
8	1.087	10.759	0.31	293.15
9	0.737	12.253	0.31	293.15
10	0.345	13.757	0.198	293.15

**Table 2:** Analyzed case conditions.

Code	Run	$P_{1}\;[\mathrm{Pa}]$	$P_{2}\;[\mathrm{Pa}]$	*T_1_* [K]	$M_{1}\;[-]$	$M_{2}\;[-]$	*Tu* [%]	H_d_ [mm]
4321	158	243.454	142.386	808	0.17	0.91	8.3	34

**Table 3:** Overview of analyzed viscous models (“+” used, “-“ not used, “N/A” not available).

No.	Viscous Model	Viscous Heating (VH)	Curvature Correction (CC)	Production Kato-Launder (KT)	Production Limiter (PL)	Energy Prandtl Number
1	k−ω SST	+	+	+	+	0.85
2	k−ω SST	+	+	+	+	1.00
3	k−ω SST	+	+	+	+	Wassel and Catton
4	k−ω SST	+	+	+	+	Kays and Crawford
5	transition SST	+	+	+	+	0.85
6	k−ω SST	+	−	+	+	0.85
7	k−ω SST	−	+	+	+	0.85
8	k−ω SST	+	+	−	+	0.85
9	k−ω SST	+	+	+	−	0.85
10	k−ω SST	+	+	−	−	0.85
11	Spalart- Allmaras	+	+	N/A	N/A	0.85

## Data Availability

The data presented in this study are available on request from the corresponding author. The data are not publicly available due to project restrictions.
